# A Comic History of British Nursing

**Published:** 1913-03-08

**Authors:** 


### A COMIC HISTORY OF BRITISH NURSING.

In another column (p. 622) will be found a review of the third and fourth volumes of a history of nursing upon a scale larger and more elaborate than has been previously attempted. A large section of the third volume is devoted to the story of British nursing in what may be called the period since Miss Nightingale; and a large part of that section is taken up by an account of the long controversy which centred round the question of the State registration of nurses. We have no intention of discussing here the pros and cons of that controversy and of the various side issues which arose from it. But a word of protest does seem needed concerning the way in which the account of those ancient disputes has been written.

The general editor of the work, Miss Lavinia L. Dock, has entrusted to a small group of collaborators the chapters which deal with the last thirty or forty years of nursing in Great Britain. The list is a short one, containing four names. The photograph of one of these ladies forms the frontispiece to the volume, and two of the remaining three are similarly immortalised further on. The text does not hesitate to speak of these collaborators in terms of perfervid eulogy, and to extol their views and their work and everything that they do, or want to do, in fantastic adulation. If that were all, it might pass for nothing more than an exhibition of self-centred and ill-balanced emotionalism, combined with a pitiable breach of good taste to produce that self-praise which is said to be no great recommendation. But, not content with lauding themselves and each other to the skies, the four ladies must needs belabour all those who disagree with them or oppose their projects for the improvement of the status of nurses. The basest motives are freely imputed, not only to the Editor of The Hospital, but to many others who share his views, wholly or in part. No attempt is made to look at both sides of the question; and it never seems to have struck the four collaborators that anyone can honestly and conscientiously hold opinions different from theirs.

This peculiar frame of mind may be of use for the game of mud-slinging; but it does not qualify its possessors for becoming historians. Apparently, too, the Suffragette mania has them in the toils, for certain passages distinctly imply that the opposition to registration is all part and parcel of the cowardly masculine plot to oppress and exploit the downtrodden feminine half of the community. Here, we fancy, the collaborators are making more than a hole in their manners—a tactical blunder. We do not think there is any band of professional women so indifferent to the Suffrage agitation, so resolute in condemnation of the excesses which have lately disfigured it, as the trained nurses of this country are. Be that as it may, it is quite certain they are sufficiently acute to assess at its true value a "history" of nursing, whose foundation-stone is a glaring breach of ordinary good taste, and whose leading characteristics are prejudice and back-scratching.

